# Ependymal ciliary motion and their role in congenital hydrocephalus

**DOI:** 10.1007/s00381-021-05194-9

**Published:** 2021-05-17

**Authors:** Koichiro Sakamoto, Madoka Nakajima, Kaito Kawamura, Eri Nakamura, Norihiro Tada, Akihide Kondo, Hajime Arai, Masakazu Miyajima

**Affiliations:** 1 Department of Neurosurgery, Juntendo University, 2-1-1, Hongo Bunkyo-ku, Tokyo, 113-8421 Japan; 2 Laboratory of Disease Model Research, Juntendo University Graduate School of Medicine, Tokyo, Japan; 3 Department of Neurosurgery, Juntendo Tokyo Koto Geriatric Medical Centre, Shinsuna Koto-ku, Tokyo, 136-0075 Japan

**Keywords:** Primary ciliary dyskinesia, Motile cilia, Hydrocephalus, Ciliopathy

## Abstract

**Purpose:**

Since a case of hydrocephalus in humans considered to be caused by ciliary dysfunction was first reported by Greenstone et al. in 1984, numerous papers on the correlation between ciliary function and hydrocephalus have been published.

**Methods:**

We reviewed the published literature on primary ciliary dyskinesia in humans causing hydrocephalus, focusing on articles specifically examining the relation between ciliary function and hydrocephalus and its treatment. In addition, the authors’ experience is briefly discussed.

**Results:**

Full texts of 16 articles reporting cases of human hydrocephalus (including ventriculomegaly) due to defects in ependymal ciliary function or primary ciliary dyskinesia observed in clinical practice were extracted. In recent years, studies on animal models, especially employing knockout mice, have revealed genetic mutations that cause hydrocephalus via ciliary dysfunction. However, a few reports on the onset of hydrocephalus in human patients with primary ciliary dyskinesia have confirmed that the incidence of this condition was extremely low compared to that in animal models.

**Conclusion:**

In humans, it is rare for hydrocephalus to develop solely because of abnormalities in the cilia, and it is highly likely that other factors are also involved along with ciliary dysfunction.

## Introduction

**Fig. 1:**
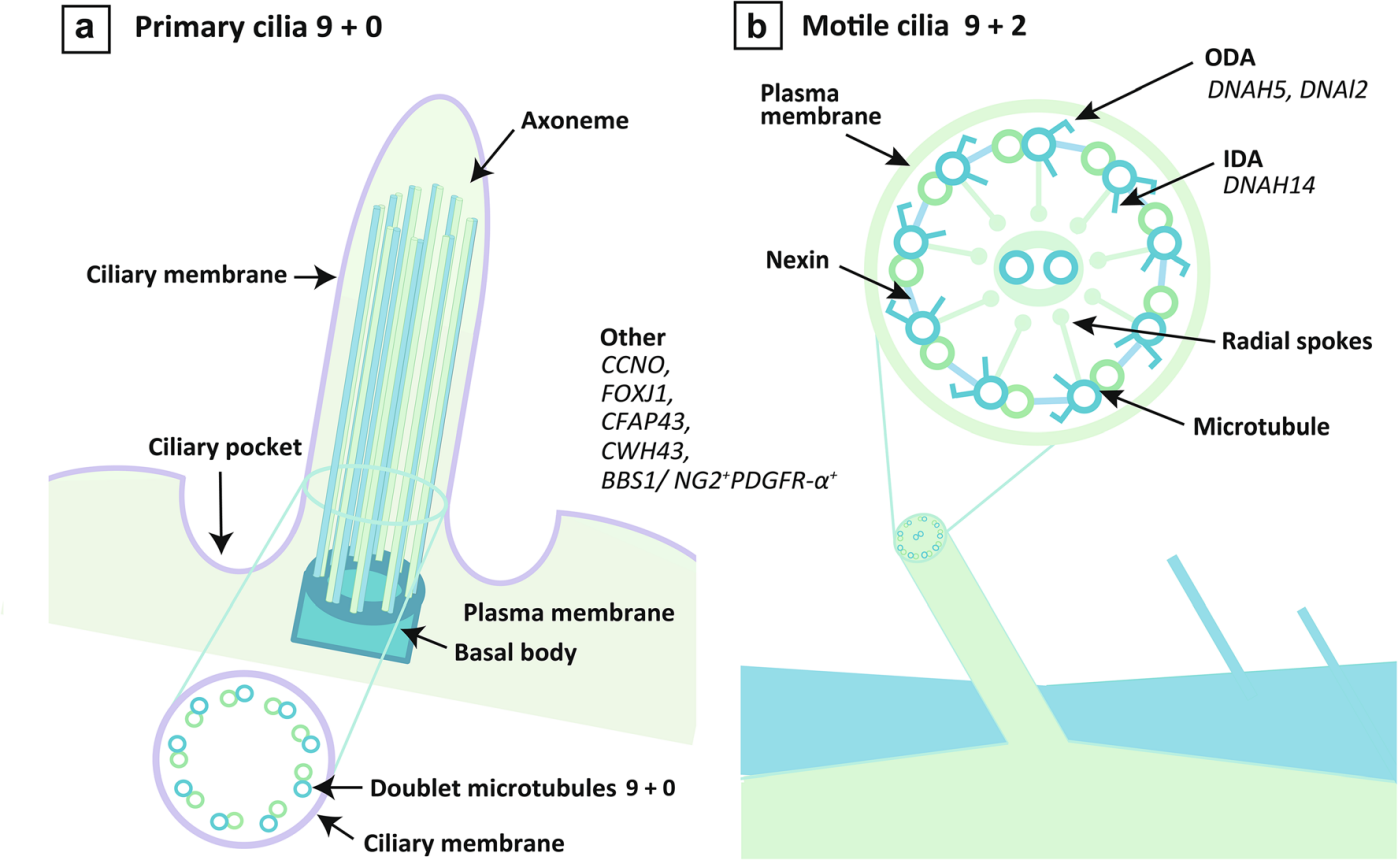
Schematic of cilia and genes reported to cause primary ciliary dyskinesia with hydrocephalus in human. Microtubules have a hollow capillary structure, in which 13 fibers are formed (**a**). The radial spokes, which are T-shaped structures inside the axoneme, and central microtubules involved in dynein arm control (**b**). Abbreviations: IDA, inner dynein arm; ODA, outer dynein arm

Cilia are hair-like structures that protrude from the cell surface of eukaryotic cells. They vary in thickness, starting at approximately 200 nm, and their length can vary from several millimeters to several centimeters. Each cilium has a central structure that is called an axoneme and contains a dimer doublet of nine ring-shaped microtubules called peripheral microtubules (PMTs). Microtubules have a hollow capillary structure, in which 13 fibers are formed by alternately arranging and polymerizing α- and β-tubulin in a cylindrical shape (Fig. 1a).

Cilia can be broadly divided into motile and primary, which can be further divided into rotating embryonic nodal motile cilia and immotile cilia in the fetus. In motile cilia, there are structures that resemble arms; the outer one is called the outer dynein arm (ODA) and the inner one, the inner dynein arm (IDA). Motile cilia have two central microtubules and nine pairs of peripheral microtubules that are arranged as nine doublets around them in a so-called 9 + 2 structure that moves on a plane resembling a whip. Those without central microtubules, such as primary cilia, are called 9 + 0 structures. There are no dynein arms in the immotile cilia. Each PMT has a nexin link and radial spoke structure, connecting adjacent PMTs, which is backed by various cytoskeletons. ODA is composed of heavy, intermediate, and light chains. The dynein heavy chain possesses ATPase activity. The radial spokes, which are T-shaped structures inside the axoneme, and central microtubules are involved in dynein arm control (Fig. 1b). The central microtubule is considered to inform the dynein arm of the inclination of the cilia through the radial spoke. Dynein provides motility, and structures such as central microtubules and radial spoke define motility in a certain direction [1].

Hydrocephalus is a central nervous system disorder caused by the retention of cerebrospinal fluid (CSF) resulting from either overproduction or impaired absorption of CSF [2, 3]. Hydrocephalus can occur at all ages and is classified as congenital, pediatric, or adult according to the time of onset. Depending on its etiology, it can be considered hereditary, idiopathic, or secondary hydrocephalus [4]. To date, various types of hereditary hydrocephalus have been investigated to elucidate the pathophysiology of this disease. Most cases present with syndromic hydrocephalus, which is accompanied by malformations of multiple organs. Since the report of model rats exhibiting hydrocephalus caused by a defect in ventricular ependymal cilia in 1998, many animal models of hydrocephalus due to ciliary dysfunction have been reported [5–10]. This paper reviews and discusses the pathophysiology of hydrocephalus due to ciliary dysfunction.

## Methods

The present review was conducted according to the PRISMA statement criteria. The literature search included research published from January 1, 1984, to March 18, 2021. We included only original papers published in PubMed-indexed peer-reviewed journals, clearly stating “cilia and hydrocephalus” or “primary ciliary dyskinesia and hydrocephalus” applied. The exclusion criteria were as follows: papers not describing original research and reviews (i.e., perspectives, letters to the editor, commentaries, and abstracts), non-English–language papers, or without application to a neurosurgical field.

The search was performed using the Boolean logic of the advanced search of the PubMed database and by scanning the reference lists of the retrieved articles. Of the studies collected, the “human subjects” term was added as an inclusion criterion.

## Results

A PubMed search yielded 260 items. Among the collected studies, 244 were excluded because they met the exclusion criteria. Full texts of 16 articles reporting cases of human hydrocephalus (including ventriculomegaly) due to defects in ependymal ciliary function or primary ciliary dyskinesia (PCD) in clinical practice were extracted (Table 1).

**Table 1:** Ciliary dysfunction with hydrocephalus in humans

References	Gene	Age at the time of diagnosis	Number of people/sex	Sporadic/familial	Ciliary structure defect	Type of hydrocephalus	Comorbidity
Greenstone (1984) [11]	Not investigated	12 years	Male	S	Outer dynein arm	Obstructive hydrocephalus	Respiratory tract infection
Jabourian (1986) [12]	Not investigated	15 years	Female	S			Kartagener syndrome
De Santi (1990) [13]	Not investigated	Newborn	Female	S		Aqueductal stenosis	Respiratory tract infection, suspected PCD
Picco (1993) [14]	Not investigated	Newborn	Female	S	Inner and outer dynein arms		
Al-Shroof (2001) [15]	Not investigated	Adult	4, male	F	Inner dynein arm	Mild hydrocephalus	PCD
Wessels (2003) [16]	Not investigated	Newborn	3	F	Deficiency of the IDA, radial spokes and nexin links	Mild ventriculomegaly	PCD, Kartagener syndrome
Kosaki (2004) [17]	Not investigated	Prenatal	3 (all deceased)	F	Inner dynein arm	Severe hydrocephalus	Trilobed left lung
Vieira (2012) [18]	Not investigated	Newborn	Female	S	Inner and outer dynein arms	Aqueductal stenosis	A complex heart malformation (dextrocardia, common atrium, and patent ductus arteriosus), PCD
Berlucchi (2012) [19]	Not investigated	8 years	Female	F	Total absence of cilia in epithelial cells	Mild hydrocephalus	Respiratory tract infection, PCD
Kageyama (2016) [20]	*DNAH14*	Adult	2, female	S	Inner dynein arm	Communicating hydrocephalus	
			1, male	S			
Amirav (2016) [21]	*CCNO*	Adult	Male	S	Reduced generation of multiple motile cilia	Mild hydrocephalus	Respiratory tract infection, PCD, situs inversus
Morimoto (2019) [22]	*CFAP43*	Adult	Mother and three siblings	F	Abnormal 10 or 8 + 2 peripheral microtubules, compound cilia	Familial NPH	Respiratory tract infection, PCD
Wallmeier (2019) [23]	*FOXJ1*	Adult	6	S	Generation of motile ependymal cilia, defects of microtubular organization or missing central tubules	Obstructive hydrocephalus	Respiratory tract infection, PCD, situs inversus
Robson (2020) [24]	*MCIDAS*	Newborn and children	7	F	Reduced generation of multiple motile cilia	Ventriculomegaly	Respiratory disease
Rocca (2020) [25]	*DNAI2*	Newborn	Female	S	Outer dynein arm	Ventriculomegaly	Situs inversus
Yang (2020) [26]	*CWH43*	Adult	3	S	Ependymal cell dysfunction	Familial NPH	

*NPH*, normal pressure hydrocephalus; *PCD*, primary ciliary dyskinesia

PCD develops in approximately 1 in 15,000–30,000 people, but in countries without a specialized diagnostic system, it is treated as an undiagnosed disease while being followed. As such, there is a high possibility that this condition is underdiagnosed [27]. Because of ciliary motility and sperm flagellar dysfunction in various organs, clinical symptoms such as respiratory failure at birth, recurrent pneumonia in infancy, sinusitis, bronchitis, infertility, and serous otitis media occur owing to a gene mutation. The association between respiratory distress and the development of hydrocephalus has been described in certain strains of mice [7, 8, 28–30]. This suggests that the role of cilia covering the ependyma of the brain/spinal cord could be to ensure normal CSF flow.

Regarding the initial clinical observations, in 1984, Greenstone et al. first reported hydrocephalus associated with ciliary movement in a child with bacterial pneumonia and intubation management immediately after birth; the child developed hydrocephalus 2 weeks after birth. Jabourian et al. reported a later case in 1986. The authors described the case of a 15-year-old girl with Kartagener syndrome and hydrocephalus, and it was considered that hydrocephalus was related to dysfunction of the ciliary flow secondary to ependymal ciliary abnormalities [12]. De Santi et al. collected nasal and bronchial epithelium from children who developed hydrocephalus at 3 weeks of age. In all samples from the nasal and bronchial mucosa of the affected child, ciliated cells were replaced with abnormal respiratory ciliated cells. Ultrastructural inspection revealed morphological features consistent with PCD [13]. Kartagener syndrome, characterized by visceral inversion, chronic sinusitis, and bronchiectasis, occurs in approximately half of the patients with PCD and is presently regarded as a type of PCD [31]. Similar sporadic cases have been reported in recent years, and it has been indicated that hydrocephalus is associated with PCD/Kartagener syndrome due to dysfunction of the dynein arms of motile cilia [14, 18].

Later, al-Shroof et al. reported familial PCD as a familial disease that was manifested as a recurrent respiratory illness that occurred in nine relatives in three generations [15]. Delays in mental development were noted among four male relatives. PCD was diagnosed using microscopic examination of the nasal mucosa in three of these individuals. There were IDA impairments in a few tubules in all cases. Mild hydrocephalus was confirmed in all four male patients. There have been two reports of similar familial cases [16, 17] of varied severity, one involving mild ventriculomegaly immediately after birth and the other involving fatally severe perinatal hydrocephalus. Electron microscopic examination has revealed that dysfunction of the IDA is involved but has not elucidated patient gene mutations. Different findings were reported by Berlucchi et al. regarding hydrocephalus caused by the ciliary dysfunction. The author described that the oldest of two siblings with symptoms of PCD had mild ventriculomegaly; transmission electron microscopic examination of the nasal mucosa revealed the total absence of cilia in the epithelial cells [19].

Due to advances in research technology, the correlation between dysfunction of cilia and gene mutations has been actively studied in humans, and the results of gene analysis in human hydrocephalus cases of PCD have been reported since the latter half of the 2010s.

As follows, we introduce the available reports separately for each causative gene that has been discovered in cases where ciliary abnormalities caused hydrocephalus in humans.

### DNAH14

Kageyama et al. analyzed patients with a unique clinical entity of hydrocephalus, defined by panventriculomegaly with a wide foramen of Magendie and large cisterna magna, named PaVM. Of the 28 patients, five were younger than 10 years old, and 10 were from five families in this study [20]. Thirteen patients who were older than 60 years demonstrated gait disturbance, cognitive disturbance, or urinary incontinence, similar to idiopathic normal pressure hydrocephalus, which was recovered by a CSF shunt or endoscopic third ventriculostomy. Using copy number analysis, the authors found *DNAH14* to be a causative gene in three patients from one Japanese family with PaVM. In immunostaining of the autopsied brain, *DNAH14* was specifically localized to ependymal cells and choroid plexus epithelial cells. Since *DNAH14* encodes axonemal dynein in motile cilia, the authors suggested that *DNAH14* deletion may affect the physiological function of cilia during hydrocephalus pathogenesis.

### CCNO

Amirav et al. reported cases presenting with chronic recurrent infections of the airways which were defined as mucociliary clearance disorders by reduced generation of multiple motile cilia [21]. Mutations in two genes, Cyclin-O (*CCNO*) and multiciliate differentiation and DNA synthesis associated cell cycle protein (*MCIDAS*), have been identified as the causes of this disease [32, 33]. The authors analyzed 10 families, including 15 affected individuals, and biallelic recessive mutations were identified in *CCNO*. *CCNO* mutation was expressed by symptoms similar to those of PCD, but there were no cases with situs inversus. One of the 15 individuals presented with mild hydrocephalus. The authors concluded that the *CCNO* mutation broadly affected the generation of respiratory motile cilia and played a role in the generation of ependymal motile cilia.

### CFAP43

Morimoto et al. performed whole-exome sequencing (WES) in a Japanese family with multiple individuals who had normal pressure hydrocephalus (NPH) to identify related genes. Three of eight members among the eight siblings showed ventricular dilatation and three major symptoms of NPH, and their mother developed hydrocephalus at the age of 50 years and died at 62 years of age. They also showed recurrent respiratory tract infection and chronic sinusitis, similar to what is observed in PCD. Ultrastructural analysis of the nasal mucosa of the patients revealed abnormal cilia involving two axonemes in one ciliary shaft. Using WES, the authors found that cilia- and flagella-associated protein 43 (*CFAP43*) was a causative gene of NPH. Subsequently, the authors generated *Cfap43*-/- mice to confirm the effect of the truncation in *CFAP43* on the pathogenesis of hydrocephalus. *Cfap43*-/- mice showed ventricular dilatation, while *Cfap43*+/- mice showed no obvious differences from the wild-type mice. Immunofluorescent analysis showed decrease in the number of acetylated tubulin in the choroid plexus and *Spef2* and *Rsph4a* in epithelial cells of the lateral ventricle and trachea in *Cfap43*-/- mice. These proteins are markers of cilium mortality, the axoneme central pair, and radial spokes. Ultrastructural analysis showed abnormal 8 or 10 + 2 peripheral microtubules and compound cilia in tracheal cilia in *Cfap43*-/- mice corresponding to those found in human cases. Since the mutations are heterozygous, PCD is usually inherited in an autosomal recessive manner; the authors suggested that the C-terminal domain of *CFAP43* may be important for ciliary function [22].

### FOXJ1

Wallmeier et al. identified an autosomal-dominant cause of a distinct motile ciliopathy related to defective ciliogenesis of the ependymal cilia in six individuals using whole-exome and whole-genome sequencing. Heterozygous mutations in *FOXJ1*, which encodes forkhead transcription factors important for ciliogenesis of motile cilia, cause motile ciliopathy characterized by hydrocephalus, respiratory disease, and randomization of left/right body asymmetry. In five of the six affected individuals, obstructive hydrocephalus was detected within the first few weeks of life, which required immediate treatment by insertion of a ventriculoperitoneal shunt (VPS). In one remaining individual, obstructive hydrocephalus was detected at the age of 54 years; and therefore, the placement of VPS was recommended.

*Foxj1* has been associated with motile cilia formation in mammals. Mice lacking *Foxj1* activity show reduction or loss of motile cilia in many tissues, suggesting that this gene plays a fundamental role in the generation of motile cilia.

The authors emphasize that the pathophysiological link between the development of hydrocephalus and a severe mucociliary clearance disorder should be considered in the clinical care of hydrocephalus and respiratory symptoms, and that early clinical and genetic diagnosis will aid in the implementation of appropriate neurological and respiratory care in *FOXJ1*-mutant individuals [23].

### MCIDAS

Robson et al. reported that *MCIDAS*-related reduced generation of multiple motile cilia (RGMC) had a high incidence of hydrocephalus, arachnoid cysts, and choroid plexus hyperplasia (CPH).

In the article, the authors note that RGMC is a severe PCD phenotype associated with loss of motile cilia, and three mutations have been identified as the causes. *MCIDAS*, *CCNO*, and *FOXJ1* are the causes of hereditary RGMC. *MCIDAS* is an upstream regulator of human multiciliated cell differentiation. *CCNO* acts downstream on mother centriole generation and migration. *FOXJ1* which also regulates multiciliogenesis is downstream of the same pathway of *MCIDAS* and in a distinct pathway that works in parallel with *CCNO*.

Seven cases of *MCIDAS* mutation have been reported by the Leicester UK national PCD diagnostic laboratory, and all cases showed ventriculomegaly and respiratory symptoms but no situs inversus. Therefore, it is considered that the *MCIDAS* mutation is not involved in the left–right determination.

The authors concluded that the existence of diffuse CPH in patients with *MCIDAS* mutation induces CSF overproduction and triggers secondary hydrocephalus [24].

### DNAI2

Rocca et al. reported a case of a Moroccan woman who was diagnosed with ventriculomegaly and situs inversus with fetal ultrasonography; after birth, the size of her ventricles progressed. She had symptoms of PCD, such as oxygen desaturation due to respiratory infection immediately after birth and the need for O_2_ supplementation. At 4 months, genetic diagnosis was carried out using next-generation sequencing, and a novel homozygous deletion within the dynein axonemal intermediate chain 2 (*DNAI2*) gene was identified. Transmission electron microscopy revealed ODA absence or shortening by 100% of the ciliary cross sections in the ultrastructure of the nasal epithelium. Although ventricular enlargement was observed, there were no neurological symptoms; therefore, no surgical treatment for hydrocephalus was performed.

*DNAI2* is an intermediate chain dynein of the ODA, which has been cloned and characterized using the candidate gene approach. Mutations in the *Chlamydomonas* ortholog (*IC69*) have been reported to cause an immotile mutant strain (oda6), which resulted in the loss of ODA. *DNAI2* mutations are found in 2% of all PCD families and in 4% of PCD families with documented ODA defects.

The authors suggested that the low prevalence of hydrocephalus in patients with PCD signified that the genetic mechanism differed between humans and mice, while there have been many reports of genetic abnormalities that combined PCD and hydrocephalus in animal models. They concluded that this report contributed to better delineation of the important role of *DNAI2* as causative of PCD phenotype, suggesting that the variations in *DNAI2* may be a new genetic risk factor for hydrocephalus [25].

### CWH43

Yang et al. performed WES of DNA obtained from 53 unrelated patients with idiopathic NPH who responded to CSF shunting, and two recurrent heterozygous loss-of-function deletions in cell wall biogenesis 43 C-terminal homolog (*CWH43*) were observed in 15% of the patients. *CWH43* mutant mice generated in that study developed communicative hydrocephalus at 6 months of age. Immunohistochemistry for *Cwh43* showed a loss of *Cwh43* immunoreactivity in the ventricular epithelia of *CWH43* mutant mice. Ultrastructural analysis showed a decrease in the number of ependymal cilia by approximately 28% in homozygous mutant mice and 25% in heterozygous mutant mice. The authors showed that loss of *Cwh43* expression resulted in a decreased association of the GPI-anchored proteins, such as RFP-fused folate receptor alpha and CD59, with intracellular vesicles using generated HeLa cell lines containing truncated *Cwh43* protein. In agreement with these findings, the *CWH43* mutation resulted in the decreased association of CD59 with the lipid microdomain fraction of the multiciliated choroid plexus and ependymal cells in both homozygous and heterozygous CSH mutant mice. Based on these findings, the authors hypothesized that mislocalization of GPI-anchored proteins in the multiciliated choroid plexus and epithelial cells disrupted the normal function of these cells [26].

## Discussions

PCD is a congenital disease caused by mutations in genes that control normal cilium function. However, it was estimated that the identified genes accounted for only 60–70% of PCD cases, and the cause of the remaining 30% was unknown [34]. Hydrocephalus associated with PCD has been widely reported in animal models, including primarily in knockout mice. The occurrence of PCD and hydrocephalus has been reported in the past and has been shown to be associated with mutations in various genes (*DNAI1*, *DNAH5*, *DNAH11*, *DNAI2*, *KTU*, and *RSPH9/4A*) [35, 36].

**Fig. 2:**
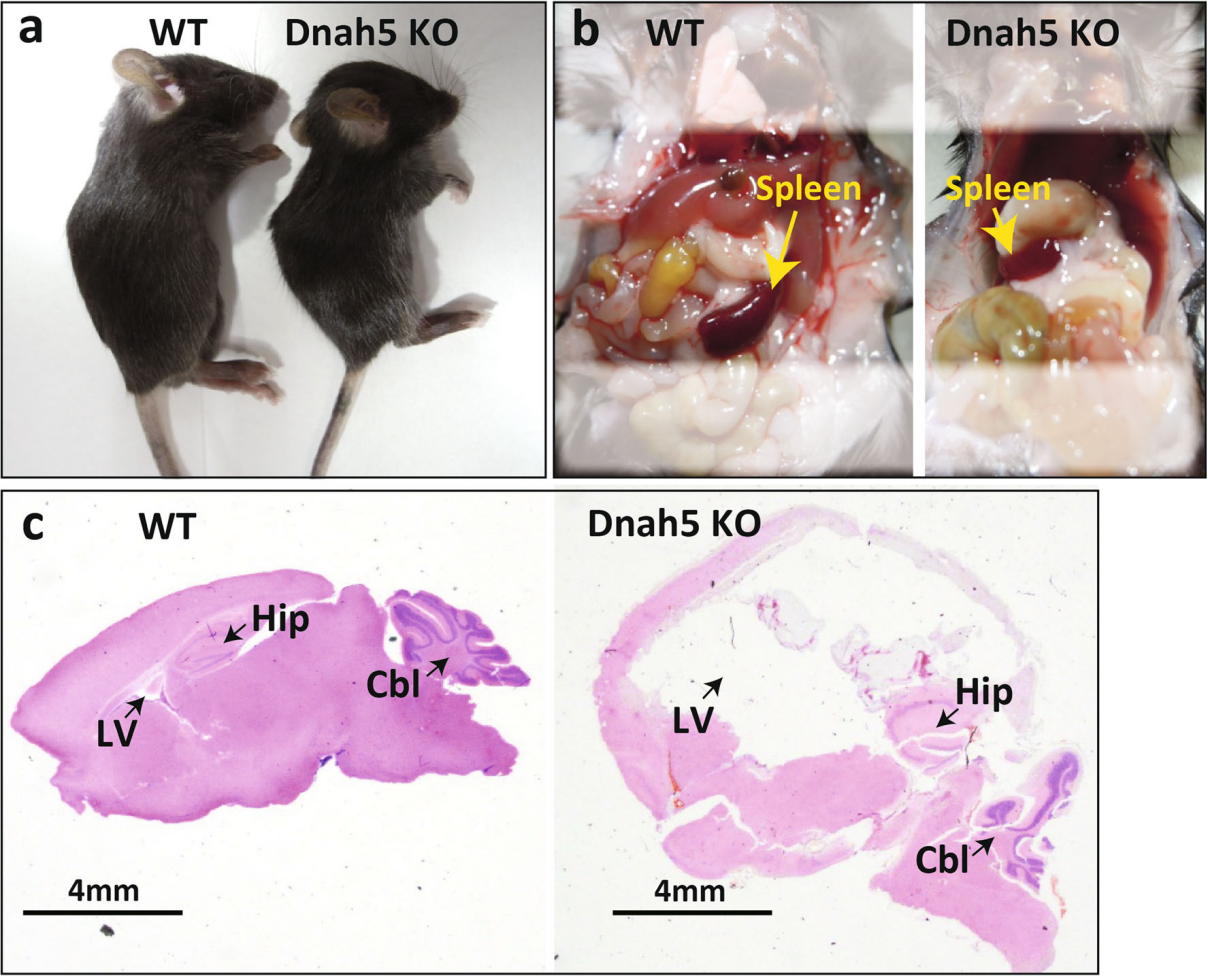
Dynein axonemal, heavy chain 5 (*DNAH5*) knockout mouse. Genetically modified animal models with *DNAH5* knockout (KO) mouse technique in our laboratory: sgRNA containing the target sequence of *DNAH5* exon2 and Cas9 protein were mixed and injected into the cytoplasm of C57BL/6J mouse–derived prenuclear stage eggs using a piezoelectric impact drive unit. The results of the experiments with knockout mice–targeting exon2 of *DNAH5* in 3 weeks after birth showed the external appearance (**a** left, wild type; **a** right, *DNAH5* KO), with visceral inversion (**b** left, wild type; **b** right, *DNAH5* KO), and with developed hydrocephalus (sagittal section, **c** left, wild type; **c** right, *DNAH5* KO). Abbreviations: Cbl, cerebellum; Hip, hippocampus; LV, lateral ventricle; WT, wild type

Ibañez-Tallon et al. showed that *DNAH5*-deficient mice develop severe hydrocephalus soon after birth, which has been shown to be associated with ODA dysfunction [7]. It has been hypothesized that ODA dysfunction causes changes in the motility of ependymal cilia in turn resulting in hydrocephalus. Omran et al. reported a case of respiratory symptoms and visceral inversion in a family of Arabian descent. Genetic testing indicated an abnormality in *DNAH5*, and they identified a PCD locus on chromosome 5p [37, 38]. Since then, studies on the genetic analysis of the disease have progressed, and reports of solitary and familial forms of the disease as well as reports of detailed test results given the advances in genetic testing methods have been performed [39–45]. Hydrocephalus was associated with *DNAH5* mutation in a mouse model (Fig. 2). They showed that ependymal cilia-generated CSF flow through the cerebral aqueduct, which they called ependymal flow [7, 8]. Tan et al. similarly reported that some *DNAH5*-mutant mice had hydrocephalus, and 40% had heterotaxy [46]. As detailed studies of genetic mutations progressed, Fliegauf et al. used immunostaining with antibodies specific for ciliary components to identify ultrastructural defects in specific cilia as a test method to aid in the diagnosis of PCD, and this method has become widely used [47]. In a large-scale study in humans, it was reported that mutations in the *DNAH5* gene were mainly concentrated in five exons (34, 50, 63, 76, and 77) and assumed various forms such as nonsense, frameshift, splicing, and missense mutations [48–52].

Previously reported cases of ciliopathies in humans and generated mouse models have shown perinatal acute hydrocephalus [17]. Recently, there have been some reports on late-onset hydrocephalus caused by ciliary dysfunction. It has been pointed out in previous reports that humans are less likely to have hydrocephalus with ciliopathies than animal models [20, 21, 26, 33]. Therefore, it is rare for humans to have hydrocephalus due to cilia abnormalities alone, and it is highly likely that another factor is also involved.

**Fig. 3:**
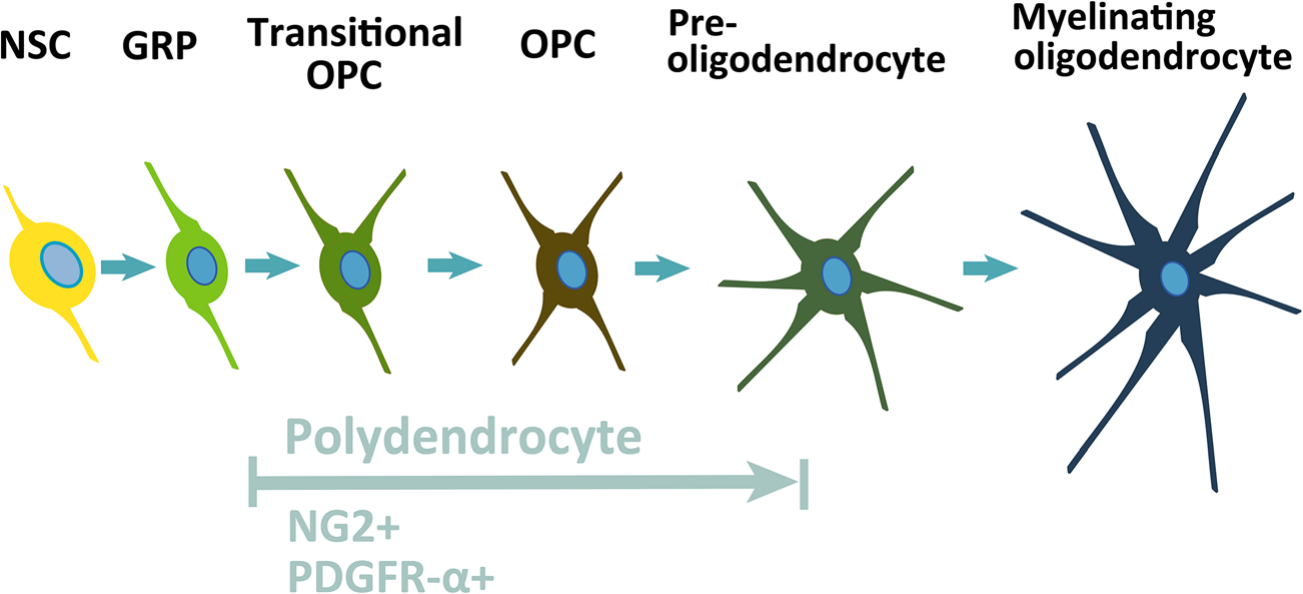
Diagram of the oligodendrocytic lineage progression: from neural stem cell to mature myelinating oligodendrocyte. Schematic depiction of the oligodendrocyte lineage identified by the antigenic phenotype in the adult mouse brain, spanning from neural stem cell (NSC), glial restricted progenitors (GRPs) to mature, myelinating oligodendrocytes, and passing through “transitional” oligodendrocyte precursor cells (OPCs), OPCs, preoligodendrocytes and premyelinating oligodendrocytes

Abnormal differentiation of NG2^+^ (platelet-derived growth factor receptor α^+^) PDGFR-α^+^ neural progenitor cells has been shown to cause congenital hydrocephalus in a mouse model of ciliary-related disease (Fig. 3). Carter et al. investigated the association between nervous system progenitor cells and hydrocephalus using a mouse model of Bardet–Biedl syndrome (BBS), a human autosomal dominant genetic disorder.

They had previously reported that BBS1-mutant mice (BBS1^M390R/M390R^) had ventricular enlargement, as seen in human BBS, but closer examination revealed that it caused ventricular enlargement between 1 and 3 days of age. Since the motile cilia of the ventricular ependyma mature 5–10 days after birth, it is likely that the appearance of ventricular enlargement in BBS-mutant mice occurs independently of the function of the motile cilia. In addition, no other causes that could result in hydrocephalus, such as obstruction of the ventricular system, abnormal choroid plexus, or properties of CSF, were found [53]. Compared with normal mice, in BBS-mutant mice, apoptosis was found to occur twice as often, and cell proliferation was found to occur approximately half as often. They carefully investigated what types of periventricular cells had abnormal cell proliferation and apoptosis and found that these were oligodendrocyte precursor cells (OLPs), a type of nervous system progenitor cells that express NG2^+^PDGFR-α^+^ (Fig. 3). The number of OLPs was low in the brains of BBS-mutant mice due to increased apoptosis and decreased cell proliferation. To investigate whether mutations in *Bbs1* in OLPs caused hydrocephalus, they created a gene-deficient mouse termed a conditional knockout which was genetically engineered to prevent *Bbs1* from being expressed in PDGFR-α^-^expressing (PDGFR-α^+^) cells. As expected, those mice always had hydrocephalus, similar to the previously mentioned mice in whom systemic *Bbs1* was mutated. In the brains of these mice, increased apoptosis and decreased cell division were also observed, most of which occurred in OLPs of NG2^+^PDGFR-α^+^. These results suggest that hydrocephalus occurs when *Bbs1* does not function in PDGFR-α^+^ neural progenitor cells. *Bbs1* controls the proliferation of nervous system progenitor cells, and PDGFα specifically binds to PDGFR-α. PDGFα is involved in the survival and proliferation of OLP. The dysfunction of the PDGFα signal was elucidated to be the underlying cause of BBS.

**Fig. 4:**
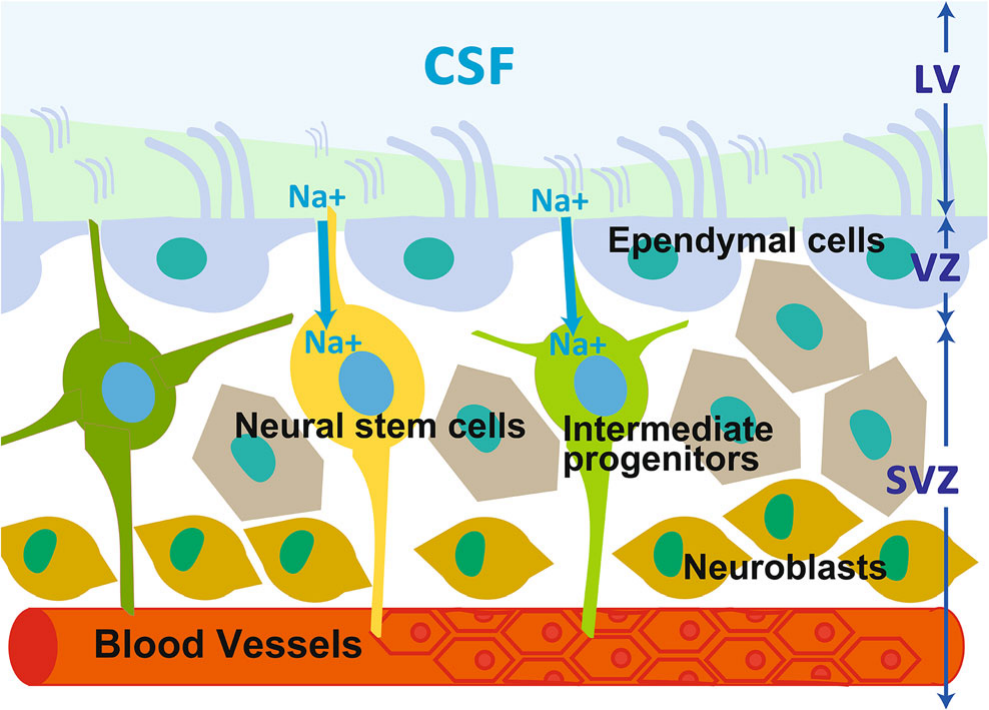
The cytoarchitecture of the neurogenic niche, consisting of the SVZ/SEZ and a monolayer of ependymal cells. The cytoarchitecture of the neurogenic niche, consisting of the subventricular zone (SVZ)/subependymal zone (SEZ) and a monolayer of ependymal cells, which have motile cilia that propel the cerebrospinal fluid (CSF). Neural stem cells extend an apical protrusion with a primary cilium that directly contacts the CSF in the ependymal layer, and a basal process that associates with blood vessels in the SVZ/SEZ. The slowly dividing neural stem cells give rise to rapidly dividing intermediate progenitors, which produce migratory neuroblasts. Abbreviations: CSF, cerebrospinal fluid; SEZ, the subventricular zone; SVZ, the subventricular zone

Nervous system progenitor cells expressing NG2 and PDGFR-α have been shown to play a key role in the pathophysiology of neonatal hydrocephalus. Thus far, dysfunction in the motor cilia of ventricular ependymal cells, overproduction of CSF, and atrophy of the brain have been shown to be the causes of communicating hydrocephalus. However, it has also been shown that survival and proliferative deficiency of nervous system precursor cells can be a direct cause. In Petrik et al., the authors elegantly combined in vivo genetic experiments in adult mice with cutting edge ex vivo experiments using acute brain slices and whole-mount ventricular walls exposed to artificial CSF flow to demonstrate that neural stem cells sensed the CSF flow to control their proliferation [54, 55] (Fig. 4).

## Conclusion

In recent years, studies on animal models, especially knockout mice, have revealed genetic mutations that caused hydrocephalus via ciliary dysfunction. However, few reports on the onset of hydrocephalus in human patients with PCD have confirmed that the incidence of this condition was extremely low compared to that in animal models. In humans, it is rare for hydrocephalus to develop solely because of abnormalities in cilia, and it is highly likely that other factors are also involved along with ciliary dysfunction.
